# Influence of uridine diphosphate-glucuronosyltransferases (1A9) polymorphisms on mycophenolic acid pharmacokinetics in patients with renal transplant

**DOI:** 10.1080/0886022X.2018.1489285

**Published:** 2018-07-17

**Authors:** H. S. Ciftci, E. Demir, M. S. Karadeniz, T. Tefik, I. Nane, F. S. Oguz, F. Aydin, A. Turkmen

**Affiliations:** a Department of Medical Biology, Istanbul Faculty of Medicine, Istanbul University, Istanbul, Turkey;; b Department of Nephrology, Istanbul Faculty of Medicine, Istanbul University, Istanbul, Turkey;; c Department of Anesthesia, Istanbul Faculty of Medicine, Istanbul University, Istanbul, Turkey;; d Department of Urology, Istanbul Faculty of Medicine, Istanbul University, Istanbul, Turkey;; e Department of Medical Biology and Genetics, Faculty of Medicine, Istanbul Bilim University, Istanbul, Turkey

**Keywords:** Pharmacokinetic, renal transplantation, rejection, blood concentrations, UGT polymorphism

## Abstract

**Background:** There are differences in pharmacokinetic of mycophenolic acid among individuals. The UGT1A9 enzyme is of special interest since it is the main enzyme involved in the glucuronidation of MPA. Single nucleotide polymorphisms in the UGT1A9 gene may be responsible for individual differences in the pharmacokinetics of MPA. The aim of this study was to explain MPA pharmacokinetics in UGT1A9 1399 C > T polymorphisms in Turkish renal transplant patients.

**Patients and methods:** One hundred and twenty-five living-donor transplant recipients and 100 healthy control subjects underwent UGT1A9 1399 C > T genotyping using polymerase chain reaction–restriction fragment length polymorphism. Concentrations of MPA were determined with Cloned Enzyme Donor Immunoassay (CEDIA). Besides that, all the patients were monitored for acute rejection and graft function during the study period.

**Results:** The UGT1A9 1399 C > T CC, CT, and TT genotype frequencies among patients were, respectively, 68.0%, 23.2%, and 8.8%. The CC, CT, and TT genotype frequencies among controls were, respectively, 63.0%, 23.0%, and 14.0%. There was no significant difference between patients and controls (*p* = .480, *p* = .999, *p* = .286, respectively). At first month, respectively, through blood concentrations of MPA were significantly higher in UGT1A9 1399 C > T TT carriers than in CT and CC carriers (*p* = .046). The doses for these patients were lower at first month (*p* = .021). Acute rejection episodes were not associated with the CC vs CT or TT genotypes (*p* = .064).

**Conclusions:** Our results demonstrated a correlation between the UGT1A9 1399 C > T polymorphism and MPA pharmacokinetics among renal transplant patients. Determination of UGT1A9 polymorphism may help to achieve target of MPA blood concentrations.

## Introduction

Renal transplantation is considered the standard replacement therapy for patients with end-stage renal failure. Graft survival depends on several factors such as adequate patient tailored pharmacotherapy. Immunosuppressive drugs may result in nephrotoxicity related to the development of chronic graft dysfunction and acute rejection. Application of immunosuppressive drugs affects the improvement of the organ transplants. Low levels of immunosuppression increase rejection risk, while high levels augment the risk of adverse effects [1,2]. Thus, the search for the optimal dosage regimen of immunosuppressive drugs in patients after organ transplantation, taking into account pharmacogenetic factors influencing the efficacy of therapy, continues [3].

Mycophenolic acid (MPA) is a standard immunosuppressive drug used effectively after renal transplantation. The classical triple immunosuppressive regimen involving the combined application of mycophenolate mofetil (MMF) or enteric-coated (EC) mycophenolate sodium (MYF), a calcineurin inhibitor, and a steroid hormone has become well established with the continuous development of transplantation immunology in recent years [4].

MPA is primarily metabolized by glucuronidation of the phenolic hydroxyl group by uridine diphosphate glucuronosyl transferases (UGTs) to an inactive MPA glucuronide. UGT isoforms that are involved in the metabolism of MPA include UGT1A9, 2B7, 1A8, and 1A7 [5]. Of special interest may be UGT1A9, a major enzyme engaged in metabolism of a commonly used immunosuppressive drug, mycophenolic acid and its increased activity may reduce MPA concentration and consequently reduce the MPA-mediated immunosuppression [6]. There are great interindividual differences in gene expression and enzymatic activity of UGT in adults [7]. Girard et al. showed that UGT1A9 protein level varied by 17-fold and glucuronidation activity varied by 9.5-fold in human liver microsomes [8]. One of isoforms studied in this regard is UGT1A8 showing that UGT 1A8*3 A173 Y277 mutation significantly decreases the enzyme activity [9]. The most extensive reports in this area are related to single nucleotide polymorphism (SNPs) discovered in UGT1A1, UGT1A7, UGT1A9, and UGT2B7. The polymorphisms found in UGT1A9 are I399, M33T, −2152, −665, −331/−440, −275. UGT1A8 and 1A10 are expressed throughout the gastrointestinal track. Studies have shown that polymorphisms in the UGT1A9 gene result in functional alterations in the expression or activity of the UGT enzymes *in vitro* [10].

The UGT1A locus in humans is located on chromosome 2q37 [8]. Recently, an intonic polymorphic variant (1399 C > T) in the UGT1A9 gene was found to be associated with increased glucuronidation of UGT1A1 and UGT1A9 substrates in *in vitro* studies using human liver microsomes [11].

Single nucleotide polymorphisms (SNPs) in the UGT1A9 gene may be responsible for individual differences in the pharmacokinetics of MPA. In renal transplant patients, interindividual differences of AUC and C0 of MPA have been reported. MPA with a recommended AUC0–12 h of 30–60 mg·h·L-1 has been shown to be effective in preventing allograft rejection after solid organ transplantation. In kidney recipients, the desired therapeutic range for MPA trough concentrations is 1000–3500 ng/ml. Mycophenolic acid trough concentrations less than 1000 ng/ml are associated with a higher risk of rejection, whereas supra-therapeutic concentrations may be associated with greater toxicity. Because of MPA’s narrow therapeutic window and high inter- and intra-patient variability, therapeutic drug monitoring is routinely performed in most centers [10].

Due to combined administration of the drugs, different MPA blood levels in renal transplantation patients are observed [12]. For immunosuppressive drugs used in kidney transplantation, the effects of genetic variants related to these mechanisms on clinical outcomes have been poorly explored. There is a known relationship between the effectiveness of MPA-based products and acute drug exposure in the prevention of allograft rejection [13,14]. Therefore, the aim of the study was to determine the correlation between therapeutic effects of MPA and the UGT1A9 1399 C > T polymorphism in renal transplant patients.

## Material and methods

The subjects included 100 healthy control subjects and 125 adult renal transplant patients each of whom provided written informed consent. The study included 125 kidney allograft patients (54 women and 71 men) of mean age 43.34 ± 12.13 years, who were engrafted from 2012 to 2015 in the transplantation clinics of the Medical University of Istanbul. The median follow-up of 125 renal transplant patients, who attended the clinic of Nephrology at Istanbul Medicine Faculty was 23.91 ± 12.09 months with the age range between 18 and 60. Patients who had follow up more than 3 months following kidney transplantation were included in this analysis.

### Immunosuppressive regimen

The eligibility criteria for the study were: (1) first living-donor transplantation, (2) identical immunosuppressive regimen including tacrolimus (Prograf, Astellas, Tokyo, Japan), mycophenolate mofetil (Cellcept, Roche), and steroids. Subjects had received a kidney allograft and were on an immunosuppressive regimen consisting of tacrolimus, MMF, and steroid. The renal transplant patients’ weights (in kilograms) and the daily dose (in milligrams per day) of MMF 1 year after transplantation were recorded; thus, dose per weight (milligrams per kilograms per day) could be calculated. The dosage adjustment was calculated by dividing each computation parameter by the dosage of MMF. In the MMF group, 1000 mg of MMF was administered twice a day. Tacrolimus was started at 0.15 mg/kg/dose and modified according to the patients’ trough concentrations. Target tacrolimus concentrations were 10–15 ng/mL in the first month post-transplantation, followed by a gradual decrease of the trough concentration to 5–10 ng/mL at one year and 4–6 ng/mL thereafter.

Clinical and biochemical parameters were analyzed among patients after renal transplantation in order to determine the potential risk of graft rejection. A rejection episode was defined based on clinical or biopsy findings according to the Banff criteria [15]. Clinical rejection was identified by increased creatinine levels in the absence of infection, obstruction, or evidence of drug toxicity. Acute rejection episodes were treated with a high daily dose of intravenous methylprednisolone (500 mg each dose) for 3 days; in refractory cases, with antithymocyte globulin (2.5–3 mg/kg/d) for 10–12 days. Patients were excluded if they were prescribed a medication that affected MPA blood concentrations. Acute rejection occurred in 25 (20.0%) and 19 of them [19/25 (76.0%)] were biopsy-proven acute rejection during the follow-up period. Demographic and clinical characteristics of patients are summarized (Table 1).

**Table 1.** Demographics and clinical of renal transplant patients and healthy control.

Demographic characteristics	(Mean ± SD)
Baseline demographics	
Healthy control (n)	100
Age of healthy control (n)	41.98 ± 22.08
Gender of healthy control (n) (female/male)	42/58
Number of patients (n)	125
Gender (female/male)	54/71
Age (years)	43.34 ± 12.13
Body weight (kg)	61.69 ± 11.72
Pre creatinine (mg/dL)	7.33 ± 1.82
Pre HLA antibodies: positive/negative	18 (14.4%)/107 (85.6%)
Primary kidney disease n (%)	
Chronic glomerulonephritis	54 (43.2%)
Tubulointerstitial nephritis	9 (7.2%)
Unknown	31 (24.8%)
Primary nephrosclerosis	10 (8.0%)
Amyloidosis	6 (4.8%)
Diabetic nephropathy	15 (12.0%)
Transplant characteristics	
Post-transplant follow-up (months)	26.32 ± 10.26
Hospitalization duration (months)	16.82 ± 9.74
Dialysis duration (months)	22.57 ± 12.13
Retransplantation (%)	7 (5.6%)
HLA mismatch	
0	15 (12.0%)
1–5	89 (71.2%)
>6	21 (16.8%)
Post creatinine (mg/dL)	1.22 ± 0.22
Post HLA antibodies: positive/negative	27 (21.6%)/98 (78.4%)
eGFR	83.17 ± 16.94
Acute rejection (%)	25 (20.0%)
Biopsy-proven acute rejection (%)	19 (76.0%)
Clinical rejection (%)	6 (24.0%)
Anti-rejection therapy (%)	
Steroid n (%)	6 (24.0%)
Antithymocyte globulin (ATG) (%)	16 (64%)
Steroid intravenous immunoglobulin (IVIG) (%)	3 (12%)
Induction therapy	7 (5.6%)

### Clinical data collection

Approximately on month 1 and on month 3 after renal transplantation, whole blood samples (5 mL) of the 125 patients were collected by venipuncture just prior to and 12 h after oral MMF administration. Plasma was isolated by centrifugation at 1900×*g* for 15 min and stored at −20 °C until analysis.

### Determination of MPA concentration in whole blood

The MPA concentration in whole blood was measured by cloned enzyme donor (CEDIA). immunoassay. The CEDIA MPA Assay uses recombinant DNA technology (US Patent No. 4708929) to produce a unique homogenous enzyme immunoassay system12. The assay is based on the enzyme β-galactosidase, which has been genetically engineered into two inactive fragments termed enzyme donor (ED) and enzyme acceptor (EA). These fragments spontaneously re-associate to form fully active enzymes that, in assay format, cleave a substrate, generating a color change that can be measured spectrophotometrically [16]. Use Na2EDTA or K2EDTA plasma samples. Care should be taken to preserve the integrity of the specimen from the time of collection until performance of the assay. Specimens should be labeled with both the time of blood collection as well as the last drug administration. The CEDIA MPA Assay produces a standard curve using the appropriate CEDIA MPA Calibrators. Prior to assaying patient specimens, validate assay calibration by testing control(s) with recovery ranges established for the CEDIA MPA Assay. The reportable range for the CEDIA MPA Assay is 0.3–10 μg/mL [17].

### DNA samples and DNA extraction

The whole blood samples were collected in EDTA-containing tubes. Genomic DNA was extracted by a genomic DNA purification kit (Peqlab) (Biotechnology GmbH, Wilmington, DE) according to standard protocol provided by the manufacturer. DNA concentrations were determined as 260 and 280 nm. DNA samples were stored at −20 °C until they are used.

#### Identification of genotypes of UGT1A9 1399 C ≥ T polymorphisms

Polymorphisms of the UGT1A9 1399 C > T gene were determined by polymerase chain reaction-restriction fragment length polymorphism (PCR-RFLP) as described previously. Genotyping of UGT1A9 1399 C > T was performed using PCR-RFLP assay using Forward: 5′-TATATGCCCGCCCCAGAG-3′ and Reverse: 5′-TATGTCCAGCCCAATACTAGATTTT-3′ primer pair [18]. The following PCR amplification conditions were used for UGT1A9 polymorphisms: *C1399T:* 95 °C, 4 min, followed by 35 cycles of 95 °C for 30 s, 58 °C for 60 s and 72 °C for 60 s and final 72 °C for 7 min; The PCR products for C 1399 T, UGT1A9 were digested with HpyCH4V (Fermentas). The 125 patients were then divided into three groups according to genotype of UGT1A9 1399 C > T. Group 1: patients were homozygotes with wild type for UGT1A9 1399 C > T polymorphisms (CC). Group 2: patients were heterozygous for UGT1A9 1399 C > T (CT). Group 3: mutant type for UGT1A9 1399 C > T polymorphisms (TT).

#### Statistical analysis

Data analysis was carried out using the statistical package SPSS version 16 to compute all descriptive statistics (version 21.0, SPSS, Chicago, IL). All results are expressed as the mean ± SD. The Hardy–Weinberg equilibrium was tested in the frequencies of all the genotypes using a chi-square test procedure. Daily dose and dose-adjusted MPA blood levels were expressed as mean ± SD. Between-group comparisons for the UGT1A9 1399 C > T genotypes were performed by one-way ANOVA. A multiple linear regression analysis was performed to assess the dependence between the average MPA dose-corrected through concentration and age, gender, UGT genotypes. The differences were accepted as statistically significant when *p* values were smaller than .05.

### Ethics statement

The study was performed according to the Declaration of Helsinki and with ethical approval from the ethics committee of the First Affiliated Hospital Istanbul University, Istanbul Faculty of Medicine [No:2013/91]. Written informed consent was obtained from all of the patients.

## Results

The frequency of CC genotype was 68.0% (85/125), the frequency of CT genotype was 23.2% (29/125), and the frequency of TT genotype was 8.8% (11/125). Among the healthy control subjects, the frequency of the CC genotype was 63.0% (63/100), the CT genotype 23.0% (23/100), and the TT genotype 14.0% (14/100), which was not different from the renal transplant patients: odds ratio [OR] 1.106 (95%CI 0.856–1.428), OR 1.005 (95%CI 0.762–1.325), OR 0.771 (95%CI 0.488–1.221), respectively. The frequency distribution of the genotypes was consistent with the Hardy–Weinberg equilibrium (Table 2).

**Table 2.** Frequency of UGT1A9 1399 C > T SNPs in renal transplant patients.

Genotype	n	Renal transplant patients (n:125) (%)	n	Healthy controls subjects (n:100) %	*p* value
CC	85	68.0	63	63.0	.480^a^
CT	29	23.2	23	23.0	.999^b^
TT	11	8.8	14	14.0	.286^c^
Total	100	100.0	100	100.0	

a CC versus TT/CT.

b CT versus CC/TT.

c TT versus CC/CT.

The effect of the 1399 C > T polymorphism of the UGT1A9 gene on the concentration of MPA in venous blood and renal allograft survival was evaluated. When patients were categorized into two groups i.e., wild type (CC) and mutant type (CT/TT), biochemical, baseline, and transplantation-related parameters in the mutant-type group were not significantly different from the wild-type group (Table 3). In patients carrying the mutant-type genotypes, we observed a trend of increased risk of acute rejection within 3 months after transplantation. But acute rejection was not significantly found in patients carrying CC genotype compared with patients carrying CT/TT genotypes (*p* = .064). There were no significant differences among the four groups with respect to age, gender, weight (Table 3).

**Table 3.** Comparison of MPA biochemical, baseline and transplantation-related parameters between mutant type of UGT1A9 1399 C > T SNP and wild-type.

	Wild-type	Mutant-type	*p* value
Number of patients	85 (68.0%)	40 (32.0%)	
Baseline characteristics			
Recipient gender (female/male)	35/50	19/21	.285
Body weight (kg)	60.12 ± 10.46	59.41 ± 12.75	.843
Age (yr)	44.18 ± 11.48	43.24 ± 10.49	.982
Transplantation-related characteristics			
Post-transplant follow-up (months)	27.18 ± 11.14	25.08 ± 12.22	.108
Hospitalization duration (months)	15.79 ± 10.12	16.91 ± 11.48	.649
Post HLA antibodies: positive/negative	16/69	11/29	.068
Acute rejection	16 (12.8%)	9 (7.2%)	.064
Toxicity	5 (4.0%)	8 (6.4%)	.128
Biochemical parameters			
Post creatinine (mg/dL)	1.28 ± 0.12	1.35 ± 0.35	.105
eGFR	81.12 ± 13.22	78.19 ± 15.32	.084
Steroid dose (mg/day)	5.5 ± 1.1	5.2 ± 1.6	.412
Tacrolimus daily dose (mg/kg/d) (Month 1)	0.12 ± 0.08	0.11 ± 0.06	.123
	15.44 ± 7.29	16.92 ± 7.33	.095
Tacrolimus daily dose (mg/kg/d) (Month 3)	0.11 ± 0.10	0.09 ± 0.15	.076
	6.21 ± 2.36	7.15 ± 2.48	.239

Data are presented as mean ± SD.

A comparison of pharmacokinetics parameters between different genotypes of the UGT1A9 1399 C > T polymorphism in patients receiving MPA showed statistically significant differences (Table 4). Blood concentrations of MPA were significantly higher among patients with the mutant-type genotype versus patients of the wild-type at 1 (*p* = .046) month after transplantation. We found that UGT2B7-related polymorphisms affect the dose-adjusted MPA (Table 4).

**Table 4.** Comparison of MPA pharmacokinetics parameters between mutant type of UGT1A9 1399 C > T SNP and wild-type.

Parameters	Wild-type	Mutant-type	*p* value
Month 1			
MPA daily dose (mg/kg/d)	0.11 ± 0.08	0.09 ± 0.05	.128
MPA blood concentration (µg/mL)	4.9 ± 1.98	5.5 ± 1.47	.046*
Blood concentration/dose (µg/mL per mg/kg)	44.5 ± 24.7	61.1 ± 29.4	.021*
Month 3			
MPA daily dose (mg/kg/d)	0.09 ± 0.05	0.09 ± 0.03	.745
MPA blood concentration (µg/mL)	4.8 ± 1.25	5.1 ± 0.59	.118
Blood concentration/dose (µg/mL per mg/kg)	53.3 ± 25.0	56.6 ± 19.7	.225
		OR 95%CI	
Genotypes	(N) blood concentrations (mean ± SD)	*p* value	
Month 1			
Wild-type	85	1.35 (0.81–1.58)	
Mutant-type	40	0.046^a^	
Month 3			
Wild-type	85	2.01 (0.95–2.64)	
Mutant-type	40	0.118^a^	

Data are presented as mean ± SD.

a Compared with Mutant-type genotype.

* *p <* .05.

## Discussion

UGTsare important enzymes in the metabolism of MPA [10]. These enzymes are highly polymorphic, conferring both high expression and low activity phenotypes [7,11]. Studies have evaluated the clinical significance of UGT1A9 or other UGT polymorphisms on MPA metabolism in kidney transplant recipients [19]. The majority of studies examining the relationship between UGT polymorphisms and MPA pharmacokinetics have involved UGTIA9 SNPs. These studies have been conducted primarily in kidney transplant recipients. The distribution of UGT1A9 1399 C > T genotypes was comparable between the patient and control groups in our study. Girard et al. the UGT1A9 CI399T exhibited allele frequencies of 3.6%, 61.3%, 55.0%, and 13.2%, respectively [20]. Girard et al. and Baldelli et al. reported that the frequency of UGT1A9 CI399T was lower than those in Japanese subjects (64.3%) and higher than those in Caucasian subjects (43.8%) [20,21]. In our study, results were similar to reported data from Caucasian populations. On the other hand, Xie et al. shown that the frequency of TT genotype was higher than Caucasian population [22].

MPA is a widely used in combination with calcineurin inhibitors as maintenance immunosuppressive therapy for prevention acute and chronic rejection after transplantation [23]. The clinical response to MPA exhibits large interindividual variability, which is mainly caused by differences in pharmacokinetics. Recently, there has been an increased interest in therapeutic drug monitoring (TDM) of MMF therapy to optimize the benefit/risk index of the drug. Predose trough samples of MPA are considered most convenient and economic, thereby allowing an increased use of TDM in the transplant setting. However, the added value of TDM for MMF therapy is still under debate [24].

However, a unique dosage recommendation is still currently used in clinics. The large inter-subject variations in MPA pharmacokinetics are characterized by large differences in the MPA/MPAG plasma concentrations [17]. Many UGT-associated polymorphisms have been reported to effect UGT activity and thus affect drug metabolism. The UGT1A9 enzyme identified by Girard and Kazuyuki none of them has been reported to increase MPA glucuronidation activity to the same extent as the T-275 A and C-2152 T mutations [11,25]. Previous studies assessed the effect of allelic variants of the UGT1A9 gene in drug metabolism showing, for example, that gene promoter polymorphisms can affect its expression level, increasing or decreasing MPA exposure [26–28].

In this work, we found that the UGT1A9 1399 C > T polymorphisms were associated with MPA pharmacokinetics in renal transplant patients. Our results also showed that polymorphism of UGT1A9 1399 C > T could influence the pharmacokinetic parameters in different post-transplant early periods in Turkish renal transplant patients. In this study, the UGT1A9 1399 C > T effect the dose- adjusted through blood concentration of MPA during the post-transplantation period. However, our results showed that MPA daily doses were lower in the patients with TT mutant genotype at first month after transplantation. In this work, we found that UGT1A9 1399 C > T polymorphism was associated with MPA pharmacokinetics in Turkish renal transplant patients.

Among UGT1A9 gene polymorphisms that could also modify the response to MMF are I399C > T, M33T, −2152C > T, −665, −331/−440, and −275T > A variants [11,29]. On the other hand, Xie et al. reported that there were also no significant differences in either daytime and night time pharmacokinetics of MPA among UGT1A9 CC, CT, and TT genotype groups [22].

Mazidi et al. classify the UGT1A9 T-275A polymorphism as wild type and mutant type in their study. Mazidi et al. reported that patients carrying T-275A SNP had significantly lower AUC_0–12_ for MPA in comparison to the wild type [30]. Conflicting results concerning these polymorphisms have been observed in other studies, which might have resulted from the specific binding of different substrates to UGT1A9 affected by mutant alleles, different co-administered drugs and limitation of the sample size [30].

Xie-XC et al. 127 patients were genotyped for polymorphisms in UGT1A9 –1818T > C, I399C > T, −118T9/10, −440C > T, −331T > C, UGT2B7*3, IVS1 + 985A > G, −900A > G, UGT1A8*2, and UGT1A7622T > C. Also, Xiao-chun et al. reported that the *UGT2B7* 11 + 985A > G genotype is associated with the pharmacokinetics of MPA in Chinese renal transplant patients, which demonstrates the usefulness of this SNP for individualizing MMF dosing [22].

Johnson et al. reported that inn the univariate analysis, the UGT1A8*2 genotype explained 4.8% of the variability in MPA dose-corrected trough concentrations in all subjects. UGT1A8*2 was an important predictor of MPA dose-corrected trough concentrations in individuals who received concomitant tacrolimus [10].

Van Schaik et al. reported that the UGT1A9 I399 C > T polymorphism, previously shown to correlate with a 1.3-fold higher protein expression in I399TT homozygote individuals as compared with wild types, did not show a significant correlation with MPA AUC_0–12._ This is in line with data from an earlier study that investigated 80 Japanese individuals with respect to differences in MPAG/MPA AUC ratios in UGT1A9 I399 C > T carriers receiving MMF and tacrolimus [22]. Also, van Schaik et al. said that additional polymorphisms of the UGT1A9 gene could affect MPA levels, apart from gene polymorphisms that encode another phase I and II enzymes, or carriers such as UGT1A8, UGT2B7, and MRP2 [31]. Michelon et al. reported that found no association UGT polymorphism and clinical outcome [32].

In our study, we evaluated the effect of the 1399 C > T polymorphism in the UGT1A9 gene on immunosuppressive drug-dose to patients after renal transplantation and the potential risk for graft rejection based on the analysis of the clinical and biochemical parameters. A multiple linear regression analysis was performed to assess the dependence between the age, gender, UGT genotype (CC, CT, TT), serum creatinine. In our study, we also demonstrated that UGT1A9 1399 C > T polymorphism did not affect clinical outcome and biochemical parameters.

Several studies have evaluated the association between UGT polymorphisms and adverse effects. Some of these studies are retrospective, and there is a significant heterogeneity in definitions and assessment of adverse effects different regimens, and time after transplant [33,34]. Ting et al. reported that no association was found between diarrhea that occurred between month 3 and month 12 after transplant and AcMPAG concentrations measured at 3 months [33]. Michelon et al. examined the relationship between UGT2B7 and UGT1A9 polymorphisms and adverse effects one-year post-transplantation in 218 kidney transplants [32]. Johnson et al. showed that None of the tested variables were important determinants of MPA dose-corrected trough concentrations in individuals [10].

Also, the present study, the carriers of the UGT1A9 TT genotype had a higher risk ratio of acute rejection than non-carriers (CC genotype) but this polymorphism not significant associated with acute rejection. On the other hand, Pazik et al. reported that patients carrying UGT1A9 98 C > T did not have an increased risk of acute allograft rejection [28]. In contrast, Pazik et al shown that UGT1A9 98 C > T was significantly associated with diminished eGFR and proteinuria, predicting graft function deterioration [28]. MPA area below the target therapeutic window increases the risk of adverse effects, such as infection, anemia and diarrhea [12]. However, patients are under an increased risk of under-immunosuppression and acute rejection episodes. These facts increase the necessity of therapeutic drug monitoring, because novel regimens increase the risk of inadequate excessive immunosuppression [13].

## Conclusions

UGT 1A9 polymorphism can be partly responsible for interindividual differences among the stable renal transplant patients, although most of our patients had acceptable MPA plasma level. Future studies, involving both genetic and clinical factors in pharmacokinetics, may be useful in determining the appropriate dose of MPA after transplantation.
